# Isolation and Biochemical Properties of Type II Collagen from Blue Shark (*Prionace glauca*) Cartilage

**DOI:** 10.3390/md21050260

**Published:** 2023-04-23

**Authors:** Zhilin Pan, Baolin Ge, Mingjun Wei, Jeevithan Elango, Wenhui Wu

**Affiliations:** 1 Department of Marine Pharmacology, College of Food Science and Technology, Shanghai Ocean University, Shanghai 201306, China; 2 Department of Biomaterials Engineering, Faculty of Health Sciences, UCAM-Universidad Católica San Antonio de Murcia, Guadalupe, 30107 Murcia, Spain; 3 Center of Molecular Medicine and Diagnostics (COMManD), Department of Biochemistry, Saveetha Dental College and Hospitals, Saveetha Institute of Medical and Technical Sciences, Saveetha University, Chennai 600 077, India; 4 Marine Biomedical Science and Technology Innovation Platform of Lin-Gang Special Area, Shanghai 201306, China; 5 Putuo Branch of International Combined Research Center for Marine Biological Sciences, Zhoushan 316104, China

**Keywords:** type II collagen, blue shark (*Prionace glauca*) cartilage, microstructure, thermal stability

## Abstract

Numerous studies have shown that type II collagen (CII) has a potential role in the treatment of rheumatoid arthritis. However, most of the current studies have used terrestrial animal cartilage as a source of CII extraction, with fewer studies involving marine organisms. Based on this background, collagen (BSCII) was isolated from blue shark (*Prionace glauca*) cartilage by pepsin hydrolysis and its biochemical properties including protein pattern, total sugar content, microstructure, amino acid composition, spectral characteristics and thermal stability were further investigated in the present study. The SDS-PAGE results confirmed the typical characteristic of CII, comprising three identical α_1_ chains and its dimeric β chain. BSCII had the fibrous microstructure typical of collagen and an amino acid composition represented by high glycine content. BSCII had the typical UV and FTIR spectral characteristics of collagen. Further analysis revealed that BSCII had a high purity, while its secondary structure comprised 26.98% of β-sheet, 35.60% of β-turn, 37.41% of the random coil and no α-helix. CD spectra showed the triple helical structure of BSCII. The total sugar content, denaturation temperature and melting temperature of BSCII were (4.20 ± 0.03)%, 42 °C and 49 °C, respectively. SEM and AFM images confirmed a fibrillar and porous structure of collagen and denser fibrous bundles formed at higher concentrations. Overall, CII was successfully extracted from blue shark cartilage in the present study, and its molecular structure was intact. Therefore, blue shark cartilage could serve as a potential source for CII extraction with applications in biomedicine.

## 1. Introduction

Marine organisms have abundant resources of bioactive substances for several therapeutic applications. In this sense, several active substances such as polysaccharide, protein (especially collagen), and peptides were previously obtained from the cartilage of marine organisms, such as sharks, sturgeon, rays and stingrays. For instance, many recent studies reported the potential availability of cartilages for extraction of chondroitin sulfate from shark [1,2], antioxidant collagen peptides from Siberian sturgeon (*Acipenser baerii*) [3], type II collagen from skate and sturgeon [4,5], gelatin from Siberian sturgeon (*Acipenser baerii*) [6], collagenous polypeptide (37 kDa) prepared from whale shark cartilage (*Rhincodon typus*) [7] and low-molecular-weight protein from shark [8]. Among these biomolecules, collagen is the most abundant fibrous structural protein in animals, accounting for approximately 30% of the total protein of the human body [9]. It is widely found in the extracellular matrix (ECM) of various connective tissues (i.e., skin, bone, ligaments, tendons and cartilage) as its key structural fibrous protein [10,11]. Currently, 28 types of collagen have been identified and described, named type I to type XXVIII based on the chronological order of their discovery [9]. Of these, type II collagen (CII) is the main component of the ECM of articular cartilage and constitutes 90–95% of the total protein content in cartilage [12].

Studies have been reported on CII isolated from the cartilage of terrestrial animals (chicken, porcine, sheep, rat, zaocys and agkistrodon, etc.) [13,14,15,16,17,18] and marine animals such as Chinese sturgeon (*Acipenser sinensis*), squid, silvertip shark (*Carcharhinus albimarginatus*) and whale shark (*Rhincodon typus*) [12,19,20,21]. These studies characterized the SDS-polyacrylamide gel electrophoresis (SDS-PAGE) profiles, amino acid composition, UV spectrum, Fourier transform infrared (FTIR) spectroscopy, circular dichroism (CD) spectrum, differential scanning calorimetry (DSC) profiles, solubility and peptide mapping of CII. CII has a fibrous microstructure with a maximum UV absorption peak between 212 nm and 230 nm [16,18,21,22] and a denaturation temperature between 28.8 °C to 44 °C [12,16,19,20]. At the same time, most of the above studies have confirmed that CII holds great potential in the treatment of rheumatoid arthritis [13,18,23,24,25,26]. Research on the biochemical properties, molecular structure, conformational relationships and biological efficacy of CII is gaining attention.

Blue shark (*Prionace glauca*) belongs to *Eukarya*, *Animalia*, *Chordata*, *Chondrichthyes*, *Carcharhiniform*, *Carcharhinidae* and *Prionace*. It is widely distributed in the coastal and oceanic regions of temperate and tropical waters [27]. Blue sharks are the main bycatch target in longline fisheries and gillnet fisheries [27,28]. Surveys show that in many countries or regions, blue shark account for an average of a third or even more than half of the total catch in their large pelagic fisheries [29,30,31,32,33]. The fins of the blue shark are the traditional seafood, and its meat can be made into fish balls [27,32]. By-products from shark processing, particularly skin (10%), fin (1–3%) and cartilage (6–7%), can be used as potential source material for collagen extraction [34,35,36]. Therefore, this study aims to extract CII from blue shark cartilage and characterize its biochemical properties to provide a theoretical basis for its potential application in pharmaceuticals and medical tissue engineering.

## 2. Results

### 2.1. Protein Pattern of BSCII

**Figure 1:**
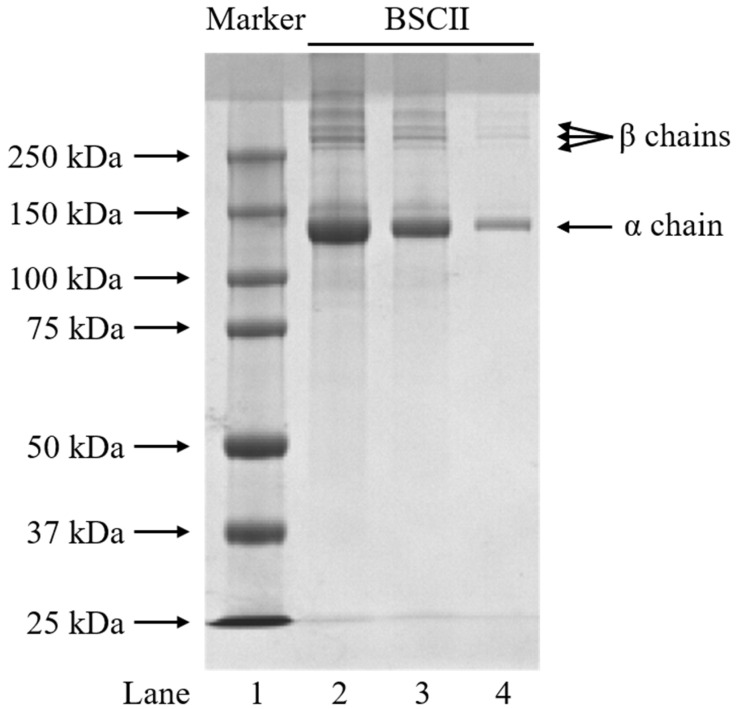
SDS-PAGE profiles of BSCII isolated from blue shark cartilage on 4.5% stacking gel and 7.5% resolving gel. Lane 1: protein markers; lane 2: BSCII (1 mg/mL); lane 3: BSCII (0.5 mg/mL); lane 4: BSCII (0.1 mg/mL).

The protein pattern of BSCII was identified using the SDS-PAGE method and the electrophoretic profile was shown in Figure 1. BSCII was comprised of an α chain and its dimer (β chains). The α chain was made up of three identical α1 chains, while β chains were formed probably due to the different number of sugar molecules attached to each α1 chain. By comparing the standard molecular weight protein marker, the molecular weight of the α chain was approximately 140 kDa. This suggests that BSCII primarily comprises type II collagen with the configuration [α_1_ (II)]_3_. As expected, the width and color depth of the bands increased with increasing protein concentration (from 0.1 mg/mL to 1 mg/mL). In addition, no non-collagenous protein bands were observed in the electrophoretic profile, indicating that the extracted proteins reached electrophoretic purity.

### 2.2. Total Sugar Content of BSCII

The total sugar content of BSCII was determined by the phenol-sulfuric acid method. The total sugar concentration of BSCII was calculated by substituting the absorbance at 490 nm of the treated 1 mg/mL BSCII solution into the equation of the glucose standard curve. Then, the total sugar content of BSCII was found to be (4.20 ± 0.03)% by conversion.

### 2.3. Microstructural Analysis

**Figure 2:**
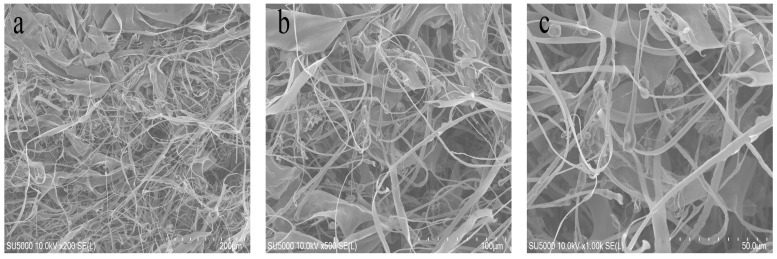
Scanning electron microscopic structure of BSCII isolated from blue shark cartilage. SEM image with different magnifications: (**a**) 200 μm, (**b**) 100 μm, (**c**) 50 μm.

The microstructural features of the freeze-dried BSCII were observed using a scanning electron microscope (SEM) at different magnifications (200, 100 and 50 μm) (Figure 2). It could be clearly seen as a fibrillar, flaky, loose and porous structure, the largest proportion of which were randomly curled fibrous filaments. This might be due to the sugar chains attached to the CII molecules that occupy certain spatial positions, resulting in collagen molecules that do not easily align together. Type I collagen, on the other hand, mostly showed a flaky structure, probably owing to the interconnection of filamentous collagen molecules [37]. This suggested that BSCII was a CII, which was consistent with the SDS-PAGE result. Furthermore, the surface of the flakes was partially wrinkled, possibly because of dehydration during drying [38].

**Figure 3:**
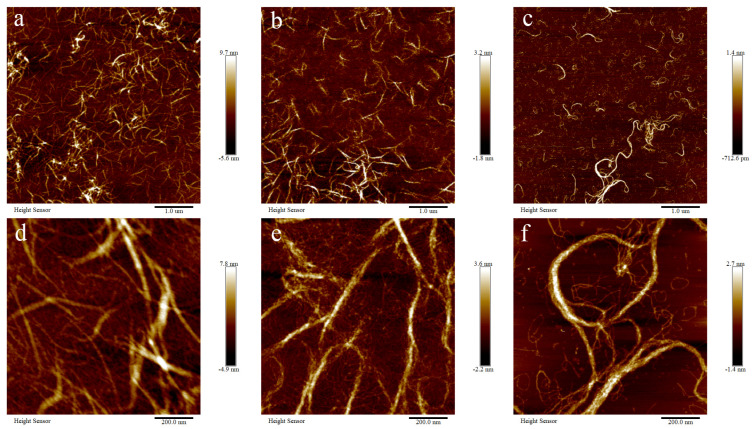
Atomic force microscopic structure of BSCII isolated from blue shark cartilage. AFM image of different concentrations of BSCII: (**a**,**d**) 1 mg/mL, (**b**,**e**) 0.5 mg/mL, (**c**,**f**) 0.1 mg/mL. AFM image with different magnifications: (**a**–**c**) 1 μm, (**d**–**f**) 200 nm.

The microstructure of BSCII solutions at different concentrations (1, 0.5 and 0.1 mg/mL) was observed using an atomic force microscope (AFM) at different magnifications (1 μm and 200 nm) (Figure 3). The fibrous structure of collagen in the solution state could be clearly observed, and the greater the concentration, the denser the fibrous bundles.

### 2.4. Amino Acid Composition of BSCII

The amino acid composition of BSCII and other biological sources of CII was shown in Table 1. BSCII had the highest glycine content, accounting for approximately 35.38% of the total amino acid content. Glutamic acid, alanine and proline contents were high, whereas the methionine, isoleucine, tyrosine, phenylalanine and histidine contents were relatively low, and tryptophan and cysteine were not detected. The content of hydroxyproline, a characteristic amino acid of collagen, was also relatively high. Furthermore, the amino acid composition of CII from different biological cartilage sources was essentially similar. However, due to the differences in species, the same amino acid residues were slightly different in number between the different species. Imino acid (proline + hydroxyproline) content had an important effect on the thermal stability of collagen [12]. As shown in Table 1, the imino acid content of BSCII was higher than that of CII from other biological cartilage sources. Rigby [39] stated that the imino acid content of fish collagens was associated with their habitat.

### 2.5. Ultraviolet (UV) Absorption Spectrum

**Figure 4:**
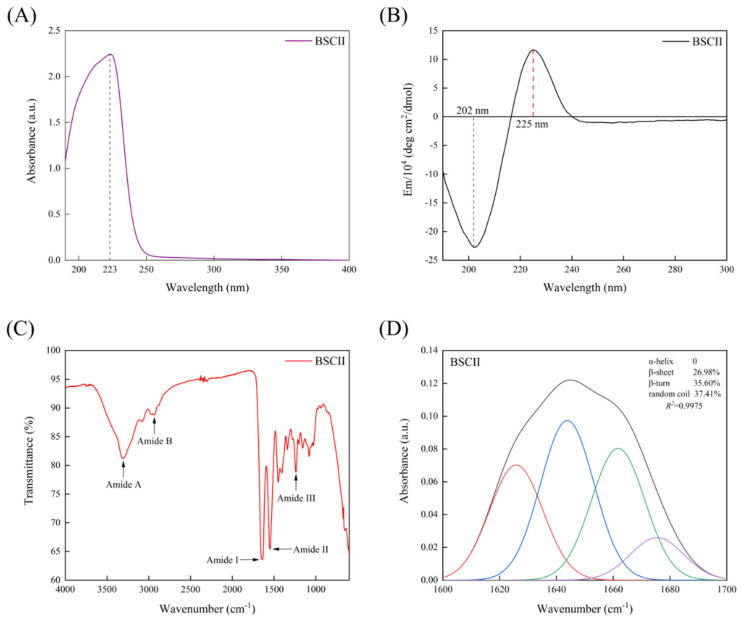
(**A**) UV absorption spectrum of BSCII isolated from blue shark cartilage. (**B**) Circular dichroism spectrum of BSCII. (**C**) Fourier transform infrared spectra of BSCII. (**D**) The secondary structure of BSCII was obtained by baseline correction, Gaussian deconvolution and second-order derivative fitting of the amide I band region (1600–1700 cm^−1^). Red line- β-sheet, blue line- random coil, green line- β-turn and purple line- β-anti.

The maximum UV absorption of BSCII was acquired at approximately 223 nm (Figure 4A), primarily due to the absorption of the C=O, −COOH and CONH_2_ groups present in polypeptide chains of collagen [40]. Most proteins had a maximum absorption peak at 280 nm, while BSCII had no significant absorption peak here. This indicated that it contained very low or even no content of tryptophan, tyrosine and phenylalanine content, which have a typical absorption peak at 280 nm [41]. It was evident that the purity of the collagen extracted by the extraction method used in this study was high, which was consistent with the SDS-PAGE result.

### 2.6. Secondary Structure Analysis

CD spectrum of BSCII was shown in Figure 4B. BSCII showed a negative absorption peak at 202 nm, typical of the irregularly curled structure of its molecular conformation, and a positive absorption peak at 225 nm, typical of the left-handed polyproline-II-helical conformation of its peptide chain [16,42].

FTIR spectra of BSCII were shown in Figure 4C. It could be seen that the most dominant absorption peaks of BSCII were at the amide band, including amide A, amide B, amide I, amide II and amide III. The peak position and causes of FTIR spectra for BSCII was shown in Table 2. In addition, the secondary structure of BSCII was analyzed using PeakFit Version 4.12 software. The results showed that BSCII contained 26.98% of β-sheet, 35.60% of β-turn, 37.41% of the random coil and no α-helix (Figure 4D).

### 2.7. Thermal Stability Analysis

**Figure 5:**
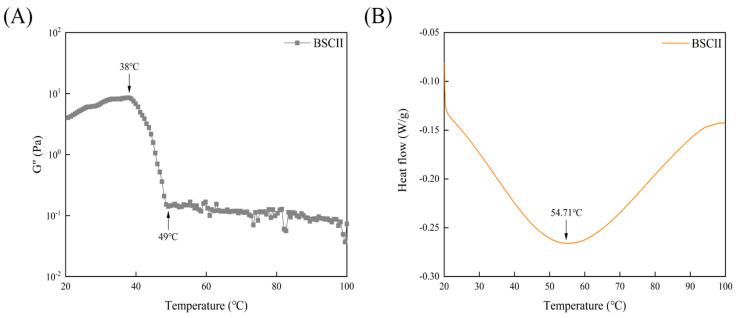
(**A**) Temperature dependence of the loss modulus (G″) of BSCII. (**B**) Differential scanning calorimetric profile of BSCII.

The variation curve of the G″ of BSCII with temperature was shown in Figure 5A. It could be seen that G″ varied little between 20–38 °C, decreasing rapidly between 38–49 °C, and remaining essentially constant from 49 °C until 100 °C. This suggested that the initial denaturation temperature (T_d_) of BSCII was 38 °C and the melting temperature (T_m_) was 49 °C. The Td temperature was determined at which the viscosity of the protein dropped by 50% [46] and hence, the Td of BSCII was 42 °C. The DSC profile of BSCII was shown in Figure 5B. There was a clear endothermic peak (54.71 °C), at which temperature the superhelical structure of BSCII was lost.

## 3. Discussion

The protein isolated from blue shark cartilage could be confirmed as CII based on the results of SDS-PAGE. As evidence, earlier studies on CII reported a similar molecular pattern of the protein [12,14,16,18,19,20,21,26]. At the same time, the three β chains suggested that the BSCII molecule might have sugar chains, which was laterally corroborated by the SEM images, and the result of the total sugar content measurement finally confirmed this conjecture.

The triple helix of collagen consisted of three molecular strands. The prolines were arranged in a left-handed polyproline-II-helical conformation, and these helices coiled together to form a right-handed superhelix. The triple helical structure of collagen could transform into a random coil when it was heated above its denaturation temperature [44]. The intensity of the positive absorption peak reflected the integrity of the triple helix structure of CII. When the triple helical structure of CII was completely disrupted, the positive absorption peak disappeared completely, and the negative absorption peak was significantly red-shifted. When CII was partially denatured, the positive absorption peak was red-shifted and the intensity decreased significantly [42,47]. In addition, the triple helical structure of collagen was more stable at higher levels of imino acid (Pro and Hyp) content as they facilitated intra- and inter-molecular cross-linking [12]. As can be seen from Figure 5B, BSCII had a complete positive absorption peak, indicating that the extraction method used in this study could retain its triple helical structure completely. The higher intensity of the positive absorption peak suggested that BSCII had a higher imino acid content, which was consistent with the results of its amino acid composition analysis.

The FTIR spectra of BSCII had similar peak positions to those of other biological CII [16,20,48], indicating that the extraction method used in this study did not cause damage to its structure. The amide A band was associated with the stretching vibration of the N−H bond. The absorption peak of a free N−H bond stretching vibration usually occurred in the range of 3400–3440 cm^−1^, and when the NH groups of a peptide chain were involved in the formation of hydrogen bonds, the absorption peak was shifted to the lower wavenumber region, usually near 3300 cm^−1^ [44]. The amide A band absorption peak of BSCII was found at 3304 cm^−1^, indicating that the N−H group of it was involved in hydrogen bonding. The absorption peak of the amide B band of BSCII occurred at 2938 cm^−1^, which was associated with the asymmetrical stretching of CH_2_ [45]. The absorption peak of the amide I band of BSCII occurred at 1633 cm^−1^. The absorption peak of the amide I band generally occurred in the range of 1600–1700 cm^−1^ and was mainly related to the stretching vibration of the C=O double bond of amide groups in proteins [46]. The absorption peak of the amide II band of BSCII occurred at 1547 cm^−1^ and was associated with the bending vibration of the N−H bond and the stretching vibration of the C−N bond [46]. Jackson et al. [46] noted that the absorption peak of collagen in 1200–1400 cm^−1^ region was an infrared spectral feature not found in other proteins, which was attributed to its unique amino acid sequence and/or structure. The high proportion of glycine and proline residues in the amino acid sequence was the most significant difference between collagen and other proteins. It is therefore reasonable to speculate that these two amino acids contribute to some extent to the spectral characteristics of the 1200–1400 cm^−1^ region of collagen. The absorption peak of BSCII at 1238 cm^−1^ was mainly attributed to the amide III band and was associated with stretching vibrations of the C−N bond and in-plane bending of the N−H. It was also attributed to the wagging vibrations of CH_2_ on its glycine backbone and proline side chains.

In addition, the absence of the α-helix in the secondary structure of BSCII was probably due to the lack of cysteine in the amino acid composition of the CII molecule. Most of the covalent cross-linking of the irregular collagen molecule occurred in its N-terminal and C-terminal non-helical regions of the peptide (telopeptides), which were excised during the enzymatic extraction of CII with pepsin, so that CII dissolved in polar solvents existed in a dispersed state [16].

G″ can reflect the viscosity of the material. For BSCII, in general, its G″ decreased with increasing temperature. This was since as the temperature increased, the degree of denaturation of collagen gradually increased, various secondary bonds within the protein molecule were broken, the molecular structure changed from a highly ordered triple helical structure to a random coil structure, the resistance to intermolecular movement decreased and the macroscopic expression was a decrease in viscosity [49]. There was a mutation between 38 to 49 °C, indicating that BSCII started to denature at 38 °C and was completely denatured by 49 °C. In contrast, the DSC results showed that the protein was completely denatured by the time the temperature reached 54.71 °C, which was consistent with the results of the temperature sweep test. The T_d_ of collagen might be influenced by the degree of hydroxylation of Pro and the Gly-Pro-Hyp sequence in it [35]. The content of imino acids had an important effect on the thermal stability of collagen. The hydroxyl group of Hyp played a major role in the stability of the triple helical structure by forming interchain hydrogen bonds [19].

## 4. Materials and Methods

### 4.1. Chemicals and Reagents

Sodium hydroxide (NaOH), ethylene diamine tetraacetic acid (EDTA), hydrochloric acid (HCl), acetic acid, sodium chloride (NaCl), methanol and ethanol were purchased from Sinopharm Chemical Reagent Co., Ltd. (Shanghai, China). Pepsin 1:3000 (enzyme activity 3000–3500 NFU/g) was purchased from Beijing Solarbio Science & Technology Co., Ltd. (Beijing, China). Phenol was purchased from Shanghai Macklin Biochemical Technology Co., Ltd. (Shanghai, China). An Omni-Easy^TM^ One-Step PAGE Gel Fast Preparation Kit (7.5%, Cat# PG211) was purchased from Shanghai Epizyme Biomedical Technology Co., Ltd. (Shanghai, China). A 5×SDS-PAGE Loading Buffer (Reducing, Cat# CW0027S) was purchased from Jiangsu Cowin Biotech Co., Ltd. (Taizhou, China). A 10×Tris/Glycine/SDS Buffer (Cat# 1610732) and Precision Plus Protein Dual Color Standards with MW of 10–250 kDa (Cat# 1610374) were purchased from Bio-Rad Laboratories Inc. (Hercules, CA, USA). Coomassie brilliant blue R250 was purchased from Shanghai Aladdin Biochemical Technology Co., Ltd. (Shanghai, China). All reagents used were of analytical grade unless otherwise stated.

### 4.2. Raw Materials and Pretreatment

The cartilage of a blue shark (*Prionace glauca*) was purchased from M/s. Yueqing Ocean Biological Health Care Product Co., Ltd. (Wenzhou, Zhejiang, China). All cartilages were stored at −80 °C for future use and thawed at 4 °C prior to pretreatment. The pretreatment of blue shark cartilage was based on the method of Phanat et al. [36] with modifications. The periosteum was removed from the cartilage. Cartilages were then cut into thin slices of 3–5 mm. The slices of cartilage were crushed using a QSJ-B02R1 meat grinder (Guangdong Bear electric appliance Co., Ltd., Foshan, Guangdong, China) and rinsed under running tap water until the ammonia odor disappeared. The cartilages were then immersed in a 0.1 mol/L NaOH at a cartilage-to-solution ratio of 1:10 (*w*/*v*) and stirred for 24 h to remove water-soluble substances, non-collagenous proteins, fat and pigments; the solution was refreshed every 8 h. Thereafter, the cartilages were rinsed with deionized water until the wash-down solution was neutral, using a FiveEasy Plus FE28 pH meter (METTLER TOLEDO, Zurich, Switzerland). The cartilages were then immersed in a 5 mmol/L EDTA solution at pH 7.5–8.0 at a ratio of 1:10 (*w*/*v*) and stirred for 40 h to remove the minerals; the solution was refreshed every 8 h. Afterwards, the cartilages were similarly rinsed with deionized water until the wash-down solution reached neutral pH.

### 4.3. Isolation of BSCII

**Figure 6:**
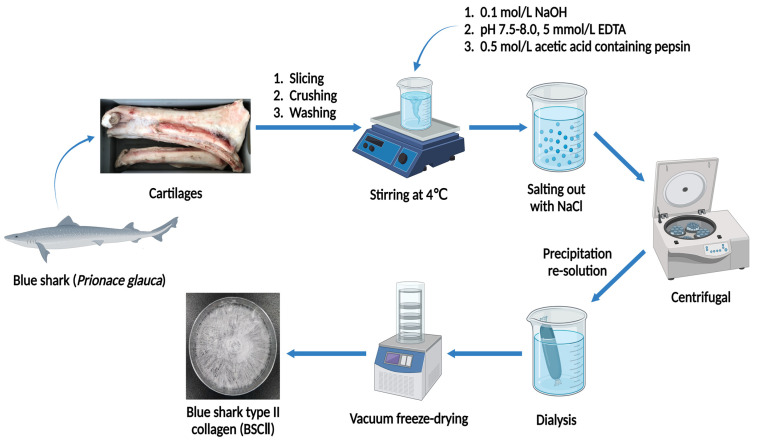
Schematic representation of BSCII extraction steps.

The isolation of BSCII was based on the method of Phanat et al. and Ge et al. [36,50] with modifications. All operations were carried out at 4 °C. The cartilages treated by the previous method were immersed in 0.5 mol/L acetic acid at a cartilage-to-solution ratio of 1:5 (*w*/*v*). Next, 0.5% (*w*/*w*) pepsin was added to the mixture of cartilage and acetic acid and stirred for 24 h. The mixture was then filtered through a double layer of gauze to obtain filtrate A. At the same time, the remaining cartilages were re-impregnated in 0.5 mol/L acetic acid containing 0.5% (*w*/*w*) pepsin at a cartilage-to-solution ratio of 1:5 (*w*/*v*) and stirred for 24 h to obtain filtrate B. The filtrates A and B were combined and centrifuged at 20,000× *g* for 10 min at 4 °C, and the supernatant was taken, using a himac CR 21G high-speed refrigerated centrifuge (Hitachi Koki Co., Ltd., Tokyo, Japan). NaCl solid was added to the supernatant to 1 mol/L for salting out and stirred at low speed until the solid was completely dissolved. Afterward, the solution was centrifuged at 20,000× *g* for 30 min at 4 °C, and the supernatant was decanted to get collagen precipitate. The collagen precipitate was then solubilized in an appropriate amount of 0.5 mol/L acetic acid with continuous stirring. The solution was transferred into some dialysis bags with MWCO of 100 kDa (Shanghai Yuanye Bio-Technology Co., Ltd., Shanghai, China) and dialyzed in deionized water at a ratio of 1:10 (*v*/*v*) until a neutral pH was obtained; the deionized water was refreshed regularly. When dialysis had been completed, the dialysate was vacuum freeze-dried using a vacuum freeze dryer (Labconco FreeZone 2.5 L, Labconco Corporation, Kansas, MO, USA). The freeze-dried collagen was named BSCII and stored in a dry place protected from light. A detailed flow chart of the BSCII preparation procedure was shown in Figure 6. The yield of collagen (dry weight) was measured from triplicate individual experiments and calculated the mean value. The yield of type II collagen was approximately 70.80–83.00 g per kg of cartilage (7.69% ± 0.61%).

### 4.4. SDS-Polyacrylamide Gel Electrophoresis (SDS-PAGE)

The molecular pattern of BSCII was determined by using SDS-PAGE. The polyacrylamide gel of 1 mm thickness comprised 4.5% stacking gel and 7.5% resolving gel was prepared using the Omni-Easy^TM^ One-Step PAGE Gel Fast Preparation Kit. Different concentrations of sample solutions (1, 0.5 and 0.1 mg/mL) were obtained by mixing different concentrations of BSCII solution (1.25, 0.625 and 0.125 mg/mL) with 5 × SDS-PAGE Loading Buffer at a ratio of 4:1 (*v*/*v*) followed by slightly oscillating, using a TGyrate Vortex Basic scroll oscillator (Tiangen Biotech (Beijing) Co., Ltd., Beijing, China). The sample solutions were heated in a metal bath (BSR-M002, Bio Medical Science Inc., Tokyo, Japan) at 100 °C for 5 min, allowed to cool to room temperature and then briefly centrifuged at 1890× *g* for 30 s, using a S1010E mini-centrifuge (SCILOGEX, Rocky Hill, CT, USA). Then, 5 μL Precision Plus Protein Dual Color Standards and 10 μL sample solution were loaded onto the gel and electrophoresed at a constant voltage of 200 V in 1 × Tris/Glycine/SDS Buffer (prepared before use) for 55 min, using a Mini-PROTEAN^®^ Tetra Cell and PowerPac^TM^ Basic Power Supply (Bio-Rad Laboratories Inc., Hercules, CA, USA). After the electrophoresis, the gel was stained on a shaker (DS-H 200, Wuhan Servicebio Technology Co., Ltd., Wuhan, China) for 30 min (shaker speed 80 rpm) in a staining solution prepared with 0.25% (*w*/*v*) Coomassie Brilliant Blue R250, 45% (*v*/*v*) methanol, 10% (*v*/*v*) acetic acid and 45% (*v*/*v*) distilled water. The gel was then transferred to a decoloring solution prepared with 20% (*v*/*v*) ethanol, 10% (*v*/*v*) acetic acid and 70% (*v*/*v*) distilled water and de-stained on the shaker until clear protein bands were observed (change the decoloring solution every hour, shaker speed 80 rpm). Finally, the gel was imaged with the GenoSens 2100 (T) Clinx Gel Documentation System (Clinx Science Instruments Co., Ltd., Shanghai, China).

### 4.5. Determination of Total Sugar Content

The total sugar content of BSCII was determined by the phenol-sulfuric acid method with reference to the method of Cuesta et al. [51] with modifications. Dried anhydrous glucose was accurately weighed to a constant weight of 2.5 mg and transferred to a 25 mL volumetric flask. Then, the volume was fixed to the scale with deionized water and shaken and mixed to obtain 0.1 mg/mL glucose standard solution. Different volumes (0, 0.1, 0.2, 0.4, 0.6, 0.8 and 1 mL) of glucose standard solutions were pipetted into test tubes and the volume was made up to 1 mL with deionized water. Three additional test tubes were taken and 1 mL of 1 mg/mL BSCII solution was added to each. Next, 0.5 mL of 5% (*w*/*v*) phenol solution and 2.5 mL of concentrated sulfuric acid were added to each of the 10 test tubes and shaken and mixed. The tubes were heated in a water bath at 100 °C for 15 min and then cooled naturally to room temperature, using a water bath (HH-S11-2-S, Shanghai CIMO Medical Instrument Manufacturing Co., Ltd., Shanghai, China). Next, 200 μL of solution from each tube was aspirated and added to a 96-well plate (Corning Incorporated, Corning, NY, USA) and the absorbance of each well was measured at 490 nm using a microplate reader (BioTek S1LFA, Agilent Technologies, Inc., Santa Clara, CA, USA). The regression equation was obtained by plotting the standard curve with the different dilution concentrations of the glucose standard solution as the horizontal coordinate and the corresponding absorbance as the vertical coordinate. The absorbance values of BSCII solution were substituted into the regression equation to calculate the total sugar concentration, which was then converted to obtain the total sugar content.

### 4.6. Scanning Electron Microscopy

Morphological characteristics of freeze-dried BSCII were visualized by a SU5000 thermal field emission scanning electron microscopy (Hitachi Koki Co., Ltd., Tokyo, Japan). A suitable number of samples were pasted onto a SEM sample holder with conductive adhesive tape and the unadhered samples were blown off with an ear wash ball. The sample holder was placed in the sample compartment of the ion sputterer for a 5 min gold ion coating and then introduced into the SEM specimen chamber. The surface morphology of collagen was observed at different magnifications (200, 100 and 50 µm) at an accelerating voltage of 10 kV.

### 4.7. Atomic Force Microscope

In order to evaluate the collagen fiber arrangement and morphological characteristics of BSCII in the solution state, the samples were observed using an atomic force microscope (Bruker Dimension Icon, Bruker Corporation, Billerica, MA, USA), following the method of Shi et al. [52] with slight modifications. Mica flake was fixed to the center of a slide with double-sided tape and the mica from the surface was removed with a clear adhesive until a flat surface was revealed. 10 μL of BSCII solution at different concentrations (1, 0.5 and 0.1 mg/mL) were cast on each of the three prepared mica flakes and dried in a desiccator at room temperature for 2 h before measurement. The samples were imaged at room temperature using ScanAsyst-Air mode scanning with a resolution of 512 × 512 pixels and a scan rate of 1 Hz. The type of silicon tip used was RTESPA-300 with a normal tip radius of 8 nm and a maximum radius of 12 nm. The nitride cantilever used had a length of 115 μm, a width of 25 μm, a thickness of 650 nm, a resonance frequency of 70 kHz and an elasticity factor of 0.4 N/m. The images obtained were treated with a “flatten” function using NanoScope Analysis 1.7 software (Bruker Corporation, Billerica, MA, USA).

### 4.8. Amino Acid Composition

The amino acid content of BSCII was analyzed by using a LA8080 Ultra High-Speed Automatic Amino Acid Analyzer (Hitachi Koki Co., Ltd., Tokyo, Japan). The amount of 13 mg of freeze-dried BSCII sample was accurately weighed and placed in a hydrolysis tube, after which 13 mL of 6 mol/L hydrochloric acid was added and the tube was sealed by blowing in nitrogen for 30 s. The tube was then placed in an electric blast drying oven (DHG-9140A, Shanghai Yiheng Technology Instrument Co., Ltd., Shanghai, China) at (110 ± 1) °C for 22 h. In the end, the tube was removed from the oven and cooled to room temperature. The hydrolysate was filtered through a 0.45 μm nylon filter membrane (Shanghai Titan Technology Co., Ltd., Shanghai, China) into a 50 mL volumetric flask, followed by rinsing the tube several times with a small amount of deionized water, and the liquid was transferred into the same volumetric flask. Finally, the volume was fixed to the scale with deionized water, shaking and mixing. Then, 1 mL of filtrate was accurately pipetted into a 3.5 cm Petri dish (Corning Incorporated, Corning, NY, USA) and dried under reduced pressure at 50 °C using a DZF-6030 vacuum drying oven (Shanghai CIMO Medical Instrument Manufacturing Co., Ltd., Shanghai, China). The dried residue was dissolved with 1.5 mL of deionized water and dried again under reduced pressure until evaporated; 1.5 mL of pH 2.2 sodium citrate buffer was added to the dried Petri dish to dissolve the residue, shaking and mixing. The solution was aspirated through a 0.22 μm nylon filter membrane (Shanghai Titan Technology Co., Ltd., Shanghai, China) and transferred to a sample vial of the instrument to obtain the sample determination solution for analysis. The amino acid analyzer was calibrated with standard reagents and positive control for all amino acids was used as a reference before the samples were analyzed. The type and content of amino acids in the sample were determined by comparing the retention time and peak area with those of the positive control amino acid peaks. The analysis of amino acid composition was replicated three times. Data were expressed as mean ± standard deviation.

### 4.9. UV Absorption Spectrum

The UV absorption spectrum of BSCII was determined using a UV spectrophotometer (UV-3000PC, Shanghai Mapada Instruments Co., Ltd., Shanghai, China) with reference to the method of Jeevithan et al. [12]. The freeze-dried BSCII sample was fully dissolved in acetic acid of 0.5 mol/L to prepare a sample solution of 0.1 mg/mL. The sample solution was placed in a quartz cuvette to determine the UV spectra absorbance from 190 to 400 nm at a scan speed of 2 nm/s with an interval of 1 nm. An equal volume 0.5 mol/L acetic acid was used as blank.

### 4.10. Circular Dichroism (CD) Spectrum

The CD spectrum of BSCII was determined using circular dichroism spectrometry (BRIGHTTIME Chirascan, Applied Photophysics Ltd., Surrey, UK) with reference to the method of Cao et al. [53] with modifications. The freeze-dried BSCII sample was fully dissolved in acetic acid of 0.5 mol/L. The solution was then transferred into a dialysis bag (MWCO = 100 kDa) and dialyzed in deionized water at a ratio of 1:10 (*v*/*v*) until a neutral pH was obtained. At the end, the sample solution of 0.5 mg/mL was obtained. An appropriate amount of sample solution was placed in a quartz cell with an optical range of 1 mm and scanned by circular dichroism spectrometry from 190 to 300 nm at a rate of 100 nm/min. An equal volume of deionized water was used as a control.

### 4.11. Fourier Transform Infrared (FTIR) Spectra

The FTIR spectra of BSCII were determined using a Fourier transform infrared spectrometer (L1050050 Spotlight 400, PerkinElmer Co., Wellesley, MA, USA), following the method of Ge et al. [50] with slight modifications. Next, 2 mg of freeze-dried BSCII was placed on the sample table of the instrument. Spectra ranging from 600 to 4000 cm^−1^ were collected in 32 scans with a resolution of 2 cm^−1^ using the attenuated total reflection (ATR) mode. The secondary structure of BSCII was analyzed using PeakFit Version 4.12 software (SeaSolve Software Inc., Framingham, MA, USA). The peak areas of each secondary structure were obtained by baseline correction, Gaussian deconvolution and second-order derivative fitting of the amide I region (1600–1700 cm^−1^). The peak area of each secondary structure was then divided by the total peak area of all secondary structures to calculate the secondary structure percentage.

### 4.12. Temperature Sweep Test

The temperature sweep test was based on the method of Moreno et al. [54] with slight modifications. The freeze-dried BSCII sample was fully dissolved in acetic acid of 0.5 mol/L. The solution was then transferred into a dialysis bag (MWCO = 100 kDa) and dialyzed in deionized water at a ratio of 1:10 (*v*/*v*) until a neutral pH was obtained. In the end, the sample solution of 5 mg/mL was obtained. The variation curve of the loss modulus (G″) of the sample solution with temperature was determined using a MCR301 rheometer (Anton Paar GmbH., Graz, Austria). The scanning temperature range was 20–100 °C and the ramping rate was 1.5 °C/min.

### 4.13. Differential Scanning Calorimetry (DSC)

DSC scanning was performed using differential scanning calorimetry (DSC 404 F1 Pegasus^®^, NETZSCH-Gerätebau GmbH, Bavaria, Germany) with reference to the method of Jeevithan et al. [12]. Then, 1 mg of freeze-dried BSCII sample was accurately weighed into an aluminum pan. Afterward, 10 μL of deionized water was added to moisten the sample and the aluminum pan was sealed with a press. The sample was scanned at 2 °C/min over the range of 20–100 °C using the sealed empty aluminum crucible as the reference.

### 4.14. Data Analysis

Each experiment was replicated three times. Data were expressed as mean ± standard deviation. All images were plotted using Origin 2019b software (OriginLab Corp., Northampton, MA, USA) except for SDS-PAGE, SEM and AFM images.

## 5. Conclusions

In this study, CII with electrophoretic purity was successfully isolated from blue shark cartilage using pepsin-restricted hydrolysis. Important biochemical and molecular structural indicators of BSCII were studied. The results showed that BSCII has the typical protein pattern, microstructure, amino acid composition, UV spectrum and FTIR spectra of CII. The extracted collagen also comprises an amount of sugar molecules, which also proves that the collagen is a glycoprotein. The secondary structure of BSCII was intact and was not altered by the extraction method used in this study. An analysis of CD spectra showed that the triple helix structure of BSCII was well-preserved. Thermal stability analysis indicated the superior thermal stability as compared to collagen from other species. All the above findings concluded that BSCII could be an excellent material for developing novel collagen-based biomaterials with great potential applications in pharmaceutical and medical tissue engineering.

## Figures and Tables

**Table 1:** Amino acid composition of BSCII and other biological cartilage sources of CII. (Data were expressed as numbers of amino acid residues/1000 amino acid residues).

Amino Acid	Sources of CII						
	Blue Shark (*Prionace glauca*) Cartilage	Chicken Sternal Cartilage [16]	Chinese Sturgeon (*Acipenser sinensis*) Cartilage [20]	Squid CARTILAGE [13]	Whale Shark (*Rhincodon typus*) Cartilage [19]	Silvertip Shark (*Carcharhinus albimarginatus*) Head Bone [12]	Silvertip Shark (*Carcharhinus albimarginatus*) Skeletal Bone [12]
Tryptophan (Trp)	N.D. ^1^	N.D. ^1^	− ^2^	N.D. ^1^	− ^2^	− ^2^	− ^2^
Aspartic acid (Asp)/asparagine (Asn)	49.04 ± 0.02	47	62	57	49.07	43.23 ± 1.75	45.76 ± 2.13
Threonine (Thr)	25.16 ± 0.02	30	23	24	26.28	23.82 ± 2.37	25.57 ± 3.55
Serine (Ser)	25.38 ± 0.05	25	51	31	32.83	37.68 ± 2.82	38.27 ± 1.81
Glutamic acid (Glu)/glutamine (Gln)	94.80 ± 0.24	94	70	90	87.43	71.88 ± 3.71	76.20 ± 2.07
Glycine (Gly)	353.83 ± 0.19	313	311	341	289.84	322.57 ± 21.06	319.69 ± 9.99
Alanine (Ala)	117.12 ± 0.62	103	104	75	96.51	135.02 ± 12.69	132.63 ± 8.46
Cysteine (Cys)	N.D. ^1^	17	3	1	2.36	7.93 ± 0.87	4.26 ± 0.94
Valine (Val)	21.26 ± 0.04	22	28	24	31.39	16.78 ± 1.35	25.43 ± 1.05
Methionine (Met)	0.42 ± 0.03	2	15	12	18.35	13.41 ± 0.74	13.54 ± 0.93
Isoleucine (Ile)	12.57 ± 0.12	13	17	18	25.70	19.59 ± 1.04	21.81 ± 2.35
Leucine (Leu)	33.38 ± 0.02	31	57	30	81.67	40.33 ± 3.93	29.95 ± 2.04
Tyrosine (Tyr)	1.86 ± 0.07	5	5	3	8.04	7.37 ± 1.44	7.19 ± 0.90
Phenylalanine (Phe)	17.66 ± 0.18	15	24	10	28.06	16.20 ± 0.55	14.97 ± 0.51
Lysine (Lys)	25.59 ± 0.05	15	17	13	25.40	22.33 ± 0.33	29.37 ± 0.77
Histidine (His)	5.57 ± 0.09	4	12	6	− ^2^	5.52 ± 0.81	9.38 ± 2.28
Arginine (Arg)	55.22 ± 0.08	53	77	56	43.12	40.94 ± 0.65	49.87 ± 1.06
Proline (Pro)	144.16 ± 0.11	94	125	96	98.83	124.29 ± 1.23	49.25 ± 1.28
Hydroxyproline (Hyp)	74.92 ± 0.09	118	− ^2^	113	58.03	51.11 ± 3.99	106.78 ± 0.79
Total	1000						
Imino acid	219.08	212	− ^2^	209	156.86	175.40	156.03

^1^ “N.D.” means not detected. ^2^ “−” means no relevant data. Values are shown as mean ± standard deviation.

**Table 2:** FTIR spectra peak position and causes for BSCII.

Region	Wavenumber (cm^−1^)	Cause	References
Amide A	3304	N−H stretch, hydrogen bond	Doyle et al. [43]
Amide B	2938	CH_2_ asymmetrical stretch	Muyonga et al. [44]
Amide I	1633	C=O stretch	Jackson et al. [45]
Amide II	1547	N−H bend coupled with C−N stretch	Jackson et al. [45]
Amide III	1238	C−N stretch, N−H in-plane bend, CH_2_ wag	Jackson et al. [45]

## Data Availability

Not applicable.

## Abbreviations

Type II collagen, CII; collagen from blue shark, BSCII; SDS-polyacrylamide gel electrophoresis, SDS-PAGE; Fourier transform infrared, FTIR; differential scanning calorimetry, DSC; circular dichroism, CD; atomic force microscopic, AFM; ultraviolet, UV; sodium hydroxide, NaOH; ethylene diamine tetraacetic acid, EDTA; hydrochloric acid, HCl; and sodium chloride, NaCl.
